# Patient-reported measures in non-invasive respiratory support for adults presenting with acute respiratory failure: a scoping review protocol

**DOI:** 10.1136/bmjopen-2026-119134

**Published:** 2026-11-09

**Authors:** Emma Duffield, Liza Keating, Sarah Wilson, Amelia Hollywood, James David van Oppen, Grace Dibben, Fiona Marie Burton

**Affiliations:** 1 School of Pharmacy, University of Reading, Reading, UK; 2 Emergency Medicine, Royal Berkshire Hospital, Reading, UK; 3 Emergency Medicine and Intensive Care Medicine, Royal Berkshire Hospital, Reading, UK; 4 University of Reading, Reading, UK; 5 Wexham Park Hospital, Emergency Medicine, Frimley Health NHS Foundation Trust, Frimley, UK; 6 Royal Berkshire Hospital, Reading, UK; 7 Centre for Urgent and Emergency Care Research (CURE), The University of Sheffield, Sheffield, UK; 8 School of Health & Wellbeing, University of Glasgow, Glasgow, UK; 9 Emergency Department, Queen Elizabeth University Hospital, Glasgow, UK; 10 College of Medical, Veterinary and Life Sciences, University of Glasgow, Glasgow, UK

**Keywords:** Patient Reported Outcome Measures, Patient-Centered Care, Hospitals, Respiratory Distress Syndrome

## Abstract

**Abstract:**

**Introduction:**

International guidance endorses patient engagement and informed treatment choices; however, research often neglects patient-reported measures (PRMs) potentially significant for patient intervention tolerance and informed decision-making. Omitting patient experience data prevents informed decision-making and limits the capture of non-physiological factors that may significantly impact treatment success. Sparse research on PRMs in non-invasive respiratory support (NIRS) may explain their absence in clinical trials. This scoping review will address this research gap by identifying PRMs used for NIRS in acute respiratory failure (ARF) in acute hospital settings.

**Methods and analysis:**

This scoping review will adhere to the Joanna Briggs Institute methodology for scoping reviews and the PRISMA-ScR reporting guidelines. Web of Science, Scopus, PubMed, Cochrane CENTRAL, Embase, PsycINFO and CINAHL will be searched for literature reporting PRMs in adults (≥16 years) receiving NIRS (continuous or bi-level positive airway pressure or high flow nasal oxygen for ARF in acute hospital settings (emergency and inpatient care). Publications will be excluded if solely community-based, lacking use of PRMs for NIRS, using other indications for NIRS, unavailable or incomplete (eg, abstracts, protocols). Although confined to the English language, no limits will exist for publication date or geographical location. Google Scholar will be searched for grey literature. Publications will be screened against inclusion/exclusion criteria and data extracted into pre-designed tables. Each step will be undertaken by two independent reviewers, with a third resolving disagreements. Data tables will be combined and findings described narratively with graphical and/or tabular displays as appropriate.

**Ethics/dissemination:**

No ethical approval is required as no original data will be used; however, ethical principles will be followed throughout. Dissemination of results will be through peer-reviewed publication and scientific conference presentations. Interested parties, such as the Royal College of Emergency Medicine, will undergo direct result-sharing.

**Trial registration:**

This project is registered with the Open Science Framework.

**STRENGTHS AND LIMITATIONS OF THIS STUDY**

- The combination of wide inclusion criteria with robust scoping review methodology (based on Joanna Briggs Institute recommendations) allows the assessment and description of a heterogeneous data set.
- A planned search of multiple prominent and relevant databases will provide a range of peer-reviewed scientific studies and minimise retrieval bias.
- An experienced research team, with multiple independent reviewers, will maximise reliability of this review’s findings.
- Due to resource limitations, the search will only include publications in English.

## Introduction

### Rationale

 Patient-centred care is central to good quality health services and results in improved health outcomes and care processes.^1 2^ Patient-reported measures (PRMs) are defined by the National Cancer Institute as ‘information about a patient’s health that comes directly from the patient’.^3^ They encompass both patient-reported experience and patient-reported outcome measures. Examples include a patient’s account of their symptoms, the impact of an illness on their physical, mental, social, emotional or spiritual well-being and their satisfaction with the care they have received. PRMs can be used to measure the impact of an intervention, to inform clinical decision-making and to evaluate care processes.

Evidence shows that engaging patients in their care and clinical decision-making can have positive effects on their health.^4^ Several studies from the USA found that patients with chronic obstructive pulmonary disease who actively engaged in planning their care had reduced mortality and longer lifespan than those who did not.^5–7^ The US Institute of Medicine’s ‘Crossing the Quality Chasm’ report urges clinicians to be ‘responsive to individual patient preferences, needs and values’.^8^ Meaningful measurement of person-centred outcomes and experiences is also a strategic objective in the UK, with the 2025 10-Year Health Plan^9^ specifying universal access for patients to choose and rate their care. Global movements like the ‘What matters to you’ campaign encourage purposeful conversations between patients and clinicians and illustrate the growing demand for the integration of patient experience and outcomes into research and clinical decision-making.^10^

Smith, the Chief Medical Officer for Scotland, champions this in his 2023–2024 annual report which promotes measuring ‘the right things including outcomes that matter to people’.^11^ He advocates for Realistic Medicine, an approach to healthcare putting patients at the centre of decisions and calling medical professionals to ensure patients have all the necessary information to make informed choices about their care.^12^ The incorporation of PRMs into research provides patient experience data to better inform treatment discussions between patients and clinicians.

Medical research aims to improve patient care but may neglect the use of PRMs within its outcomes.^13–15^ An example is the RENOVATE study: a large, multicentre non-inferiority randomised controlled trial investigating whether high flow nasal oxygen (HFNO) is as effective as non-invasive ventilation (NIV) in certain respiratory conditions.^16^ Although well-run, it did not include any PRMs within its primary or secondary outcomes. An accompanying editorial suggests incorporation of patient-centred and patient-reported outcomes, particularly around experience and satisfaction, could reveal unrecognised benefits of the interventions potentially significant for clinical decision-making.^17^ Mortality is undeniably important, but this does not detract from the influence of patient experience on treatment adherence, tolerance and success. Inclusion of the patient voice allows understanding of the acceptability of treatments, for example, is the intervention also non-inferior for the patient experiencing it?

Non-invasive respiratory support (NIRS) consists of continuous and bilevel positive airway pressure (CPAP, BPAP) and HFNO. The 2026 American Thoracic Society clinical practice guideline recommends NIRS over standard oxygen therapy when treating acute respiratory failure (ARF).^18^ NIRS can be unpleasant for patients, with estimated rates of intolerance to NIV around 10%–15%.^19^ Intolerance can result in treatment failure, escalation to intubation (and the risks of mechanical ventilation) or care withdrawal.^20^ Patient comfort is vital for therapy success, but PRM use in NIRS research is not well understood. The acute setting—emergency, acute medical (including respiratory) and critical care—is where most ARF cases are treated using NIRS. There is delivery in the community but mostly treating chronic respiratory failure in patients familiar with the therapy—some evidence shows these patients better tolerate NIRS.^21^ By focusing on the acute setting, PRM use in an unwell population who may be experiencing NIRS for the first time can be better captured.

To date, there has been no comprehensive work exploring the PRMs that exist for NIRS, how they are used, what they measure or if they are validated. This could certainly have contributed to their absence in interventional research.

This gap needs addressing: the use of PRMs in NIRS research would allow a greater understanding of non-physiological factors impacting treatment tolerance (and therefore efficacy), better inform patient-doctor discussions around treatment selection and explore potential future action to promote adherence. The first step is to understand current practice; identifying and describing the PRMs for NIRS in use clinically, and exploring how they are used, their importance and whether they are validated. The scoping review methodology allows assessment and summarisation of a heterogeneous collection of studies to describe current practice, identify knowledge gaps and inform further areas for research.

### Objective

We aim to conduct a scoping review to identify the PRMs that are currently reported in studies of NIRS in adults with ARF in the acute hospital setting. In addition, the review would describe the characteristics of these measures including domains assessed, validation status, clinical contexts, their relative importance and how they are used in the literature.

### Review question

What PRMs have been used for adults (≥16 years) receiving NIRS for ARF in acute hospital settings?

## Methods and analysis

### Study design

This review will be carried out with adherence to the Joanna Briggs Institute (JBI) methodology for scoping reviews.^22^ It is registered with the Open Science Framework online.^1^ Should any deviations from this protocol occur, these will be stated and justified in the relevant methods section of the final scoping review. The Preferred Reporting Items for Systematic Reviews and Meta-Analyses extension for Scoping Reviews (PRISMA-ScR) guidelines,^23^ with appropriate updates from the PRISMA 2020 Statement,^24 25^ will be used when reporting findings. Planned start and end dates are 20 January 2026 to 31 July 2026.

### Eligibility criteria

#### Population

This scoping review will include studies of adult human patients within the acute hospital setting who have ARF requiring NIRS. In the NHS, patients are treated on adult wards if they have completed their General Certificate of Secondary Education (GCSE) exams—this review will define adults as anyone aged 16 or over. This will include participants of all sexes, ethnicities and medical backgrounds to reflect the broad population accessing hospital care with respiratory distress. Studies of paediatric patients (aged <16 years) would be excluded. ‘Acute hospital setting’ encompasses all levels of inpatient care including emergency, acute medical, respiratory, higher monitoring and critical care wards. If a study examines a mixture of participants from the acute hospital setting and from the community (eg, on home NIV), it would also be included in this review. However, studies exclusively assessing a population using home or community-hospital-based NIRS would not be included.

#### Concept

PRMs are outcomes or experiences of treatment that are reported by the patient. NIRS consists of CPAP, BPAP (or similar bilevel methods) and HFNO. It is important to make the distinction between NIRS and NIV—the latter refers to BPAP and similar bi-level ventilation methods only. Publications will be excluded if there is no use of PRMs to monitor NIRS, or if the study subjects were not in ARF.

#### Context

Sources must be complete and accessible to the research team. Any incomplete research, such as abstracts and study protocols, along with unavailable publications will be excluded from this review. This scoping review will identify PRMs currently being used clinically, therefore those in early phases of research are unlikely to address the study question.

There will be no limitations on publication date or geographical location to keep the search as broad as possible, however sources will be confined to those published in English only. This is due to resource limitations that make translating multiple sources and languages unfeasible.

### Information sources

The databases to be searched are Web of Science (Core Collection), Scopus, Medline (PubMed), Cochrane CENTRAL, Embase (ovid.com), PsycINFO (ovid.com) and CINAHL (ebsco.com). A keyword search of Google Scholar will be conducted to identify grey literature of relevance.

The following experimental and quasi-experimental study designs would be considered: randomised controlled trials, non-randomised controlled trials, before and after studies and interrupted time-series studies. Observational studies, such as prospective and retrospective cohort studies, case-control studies, analytical and descriptive cross-sectional studies, case series and individual case reports would also be considered for inclusion along with relevant qualitative studies. Systematic reviews will undergo examination of constituent papers and reference lists, with any relevant publications considered for inclusion. Abstracts, conference papers and study protocols would not be considered for inclusion.

### Search strategy

The objective of a search strategy is to identify relevant studies that answer the review question. This scoping review will use a three-step search strategy.

First an initial limited search of Medline (PubMed) will be undertaken to identify articles on the topic. Common index terms along with frequently occurring keywords present in the titles and abstracts of relevant articles will be noted and, using a PICO-based approach, developed into a preliminary search strategy.

In the second step, the preliminary search strategy will be tailored for each database used in the search. A similarly tailored search on Google Scholar will be carried out to look for relevant grey literature. The assistance of Academic Liaison Librarian(s) in search tailoring may be used if required. See online supplemental appendix I for a proposed search strategy.

The final step involves the screening of all included papers’ reference lists to identify additional relevant publications. The reference lists and constituent papers of any relevant systematic reviews identified in the search will also be undertaken with the same goal.

### Selection of sources of evidence

All citations identified in the search will be uploaded onto EndNote V.21 (Clarivate Analytics, Philadelphia, Pennsylvania, USA) and duplicates will be removed, first using the Endnote software function, then manual checking by two reviewers. A minimum of two independent reviewers will screen titles and abstracts against the inclusion criteria. An early pilot test, where reviewers convene to clarify and resolve any issues at this stage, will be carried out as per the JBI methodology.^22^ Citations that remain following the title and abstract screening will have their full text assessed against the inclusion criteria by two or more independent reviewers. If unable to access the full texts, efforts to contact authors for paper access will be carried out with one repetition if no response comes after 1 month. Academic liaison librarians may be consulted to facilitate access to relevant publications. Any publications removed at the full text review stage will have their reasons for exclusion reported. Resolution of disagreements between reviewers at any stage will be through discussion with an additional reviewer/s. The search and inclusion process will be reported and summarised in the final scoping review using a PRISMA flow diagram adapted for scoping reviews.^23–25^

### Data extraction and charting process

Relevant data from the included publications will be extracted into a pre-developed spreadsheet data table by two or more independent reviewers in advance of data analysis. The extraction table development will involve input from the wider research team to ensure sufficient capture of all necessary metrics to address the aims of this scoping review.

Data for extraction will include general study information such as reference citation, title, author(s), publication year, geographical setting, journal, study funding, study design, clinical setting, study aims, study duration (start and end dates), study definition of ARF, eligibility criteria, control and intervention groups, participants, sample size, outcome measures, withdrawals and exclusions, length of follow-up for study as a whole and key study findings. In addition, we plan to also extract any PRM(s) reported and the study’s definitions of these, method of PRM collection (eg, verbal, written), the reported priority of the PRM(s) as outcomes if present (eg, reported as primary, secondary or tertiary outcomes or equivalent), the point(s) in the patient journey at which PRM(s) were used, PRM-specific length of follow-up, key study findings relating to PRM(s), study detail on PRM(s) validation status, study detail on PRM(s) development process (and whether this involved patient input) and any additional relevant notes.

Extraction will be carried out independently by two reviewers, and the two sets of extracted data combined and consolidated. Disagreement resolution will be through discussion or the input of an additional independent reviewer(s).

An example of a proposed data extraction table can be found in online supplemental appendix II. A pilot test on a small number of studies will be carried out by the reviewers prior to full data extraction to refine the extraction table and methods. It is the hope that a pilot test will preclude the need for any later revisions to the data extraction table. Should any alterations occur, these will be reported and justified in the final scoping review. In the case of missing data or the need for additional data, authors of papers will be contacted if appropriate.

### Critical appraisal of individual sources of evidence

This scoping review aims to map the existing literature on this topic, to identify what evidence exists and any gaps. For this reason, formal critical appraisal of individual sources will not be undertaken, though any significant methodological shortcomings may be commented on.

### Data synthesis

Data presentation will largely depend on the nature of the data collected. We anticipate the use of visual representations such as bubble plots, word clouds and/or mind maps. Graphs and tables may also display findings. Analysis will be descriptive with a narrative summary of results and how these address the study objectives and research question.

### Patient and public involvement statement

At the conception of this project, the Patient and Public Involvement (PPI) director and PPI group at the University of Glasgow were consulted regarding the requirement for a review looking into PRMs in NIRS. It was after they expressed support for this project that it was taken to the next stage of development. Another outcome of this discussion was the formation of a research steering group for anticipated follow-on work from this project, which includes a patient-member. The results of this scoping review will be reported back to the University of Glasgow PPI team.

PPI can also include partnerships that exist within the study planning process. For this project, we have partnered with a scoping review expert from the University of Glasgow whose input has been helpful in study protocol development and refining methods.

## Ethics and dissemination

As no original data is being used in this scoping review, ethical approval is not required; however, ethical principles will be adhered to throughout. Data can be freely accessed in the public domain using the methods described in this protocol.

Results will be shared through peer-reviewed publication and via presentation at scientific conferences. The University of Glasgow PPI group, Fisher & Paykel and the Royal College of Emergency Medicine will receive study findings given their involvement in the project.

## Supplementary material

10.1136/bmjopen-2026-119134 online supplemental file 1
