# Supplementary material for: Patient-reported measures in non-invasive respiratory support for adults presenting with acute respiratory failure: a scoping review protocol
Source: BMJ Open. 2026 Nov 9;16(11):e119134. doi: 10.1136/bmjopen-2026-119134 (PMC13756365; doi:10.1136/bmjopen-2026-119134)
Supplement: Supplementary file 1 [file bmjopen-2026-119134.reviewer_comments.pdf]

PEER REVIEW HISTORY

BMJ Open publishes all reviews undertaken for accepted manuscripts. Reviewers are asked to complete a checklist review form and are provided with free text boxes to elaborate on their assessment. These free text comments are reproduced below.

ARTICLE DETAILS

Title (Provisional)

Patient-Reported Measures in Non-Invasive Respiratory Support for Adults Presenting with Acute Respiratory Distress: A Scoping Review Protocol

Authors

Duffield, Emma; Keating, Liza; Wilson, Sarah; Hollywood, Amelia; van Oppen, James David; Dibben, Grace; Burton, Fiona Marie

---

VERSION 1 - REVIEW

**Reviewer**	**1**
**Name**	**Vieira , P C**
**Affiliation**	**Federal University of Uberlândia - Umuarama Campus, Intensive Care Unit**
**Date**	**27-Apr-2026**
**COI**	**None**

COMMENTS TO THE AUTHOR

The manuscript addresses an interesting topic that still presents some gaps in knowledge. The study protocol proves essential for a better study process, from the planning to the execution phase.

However, a revision of this manuscript is necessary. It is necessary to correct the overall formatting of the article, standardize the spacing between lines and before sections, punctuation rules and grammar.

SUGGESTED ARTICLES:

Grasselli G, Calfee CS, Camporota L, et al. ESICM guidelines on acute respiratory distress syndrome: definition, phenotyping, and respiratory support strategies. Intensive Care Med. 2023;49(7):727-759. doi:10.1007/s00134-023-07050-7

**Reviewer**	**2**
**Name**	**Sibanda, Elopy**

Affiliation **National University of Science and Technology Faculty of Medicine, Faculty of Medicine**

Date **21-Jun-2026**

COI **None**

The scoping review addresses an important and often overlooked component of patient care. The findings of the study should guide improvements patient care and quality of life outcomes during the difficult occasions when patients need non-invasive oxygen support. The intervention is timely and will shape patient care in the future.

Reviewer **3**

Name **Sugimoto, Hiroshi**

Affiliation **Locum**

Date **03-Sep-2026**

COI **None**

General comments:

The authors describe a protocol of scoping review, which aims to map current research on patient-reported measures (PRMs) in adult patients with acute respiratory distress requiring non-invasive respiratory support (NIRS). This protocol is generally well-designed and adheres appropriately to relevant methodological guidelines (JBI and PRISMA-ScR/P). However, several concerns need to be clarified or modified to enhance the transparency and significance of the scoping review.

Major comments:

1. Please explain the rationale for limiting the scope of NIRS to non-invasive ventilation and nasal high flow oxygen while excluding standard oxygen therapy (such as nasal cannula or non-rebreather mask). Information regarding PRMs in patients with standard oxygen therapy would also be valuable. I recommend referring the recent American Thoracic Society clinical practice guideline (PMID: 42371750).

2. Although search terms related to the "acute setting" are used in the proposed search strategy, this may narrow the search scope and risk reducing sensitivity. Furthermore, the language filter is actively used to limit the scope to English. I recommend not using the language filter.

Minor comments:

1. The authors define "adult" as someone 16 years of age or older. While the age of majority is 18 in many countries, it may be necessary to provide a rationale for including those aged 16 and older.

2. While it is stated that EndNote will be used to remove duplicate references, will tools such as Rayyan, Covidence, or generative AI be used during the subsequent process? If there is a possibility of using these tools, including this in the protocol would increase transparency.

VERSION 1 - AUTHOR RESPONSE

Reviewer: 1
Comments to the Author:
The manuscript addresses an interesting topic that still presents some gaps in knowledge. The study protocol proves essential for a better study process, from the planning to the execution phase.
However, a revision of this manuscript is necessary. It is necessary to correct the overall formatting of the article, standardize the spacing between lines and before sections, punctuation rules and grammar.

Please find structural, punctuation, and grammatical alterations made throughout the manuscript.

Reviewer: 2
Comments to the Author:
The scoping review addresses an important and often overlooked component of patient care. The findings of the study should guide improvements patient care and quality of life outcomes during the difficult occasions when patients need non-invasive oxygen support. The intervention is timely and will shape patient care in the future.

Many thanks for your positive feedback – the authors are glad that you agree this is an important area of research.

Reviewer: 3
Comments to the Author:
The authors describe a protocol of scoping review, which aims to map current research on patient-reported measures (PRMs) in adult patients with acute respiratory distress requiring non-invasive respiratory support (NIRS). This protocol is generally well-designed and adheres appropriately to relevant methodological guidelines (JBI and PRISMA-ScR/P). However, several concerns need to be clarified or modified to enhance the transparency and significance of the scoping review.

<u>Major comments:</u>
Please explain the rationale for limiting the scope of NIRS to non-invasive ventilation and nasal high flow oxygen while excluding standard oxygen therapy (such as nasal cannula or non-rebreather mask). Information regarding PRMs in patients with standard oxygen therapy would also be valuable. I recommend referring the recent American Thoracic Society clinical practice guideline (PMID: 42371750).

Many thanks for highlighting this issue. The 2026 American Thoracic Society clinical practice guideline recommends NIRS over standard oxygen therapy (SOT) in ARF

treatment. Whilst the authors agree that PRMs use should be studied across all interventions, the focus on this review is on NIRS given these recommendations and its comparatively high level of patient discomfort and consequent dependence on patient tolerance for success. This justification has been added to the Rationale section of the Introduction.

Although search terms related to the "acute setting" are used in the proposed search strategy, this may narrow the search scope and risk reducing sensitivity.

Thank you for this feedback. The acute setting was chosen as this is where most acute respiratory failure cases present and/or are treated with NIRS. It encompasses the emergency, acute medical (including respiratory), and critical care settings where the majority of NIRS is delivered. This does exclude those undergoing NIRS in the community, however many of these cases will be due to chronic respiratory failure with patients very used to undergoing these therapies – evidence shows these patients tolerate NIRS better than the general population. The justification for the acute setting is to capture PRM use in an unwell population who may be experiencing NIRS for the first time – and so can report more acutely their experience of the intervention. This reasoning has been added to the Rationale section of the Introduction.

The language filter is actively used to limit the scope to English. I recommend not using the language filter.

This is a fair criticism, and the authors agree with you. Unfortunately, resource limitations mean that translating multiple papers from other languages into English is simply not feasible. This will be discussed in the limitations section of the final review paper.

<u>Minor comments:</u>
The authors define "adult" as someone 16 years of age or older. While the age of majority is 18 in many countries, it may be necessary to provide a rationale for including those aged 16 and older.

Thank you for this feedback. In the NHS patients are treated on adult wards if they have completed their GCSE exams (which take place aged 16). As such, UK-based research often defines adult as aged 16 or over. This rationale has been added to the Eligibility Criteria section of the Methods.

While it is stated that EndNote will be used to remove duplicate references, will tools such as Rayyan, Covidence, or generative AI be used during the subsequent process? If there is a possibility of using these tools, including this in the protocol would increase transparency.

This is a valid suggestion to increase transparency. Due to the delay between initial submission at the beginning of the year and the return of reviewer comments, the searches and de-duplication of references have already happened, and these tools

were not used. Instead, the Endnote software and manual checking for missed duplicates by two reviewers was carried out. This is a matter that could be discussed in the limitations section of the final review paper.
