# Supplementary material for: Patient-reported measures in non-invasive respiratory support for adults presenting with acute respiratory failure: a scoping review protocol
Source: BMJ Open. 2026 Nov 9;16(11):e119134. doi: 10.1136/bmjopen-2026-119134 (PMC13756365; doi:10.1136/bmjopen-2026-119134)
Supplement: Supplementary file 2 [file bmjopen-16-11-s001.docx]

**Appendices**

**Appendix I: Proposed search strategy**

**Medline (PubMed):**

"Patient Reported Outcome Measures"[Mesh] OR "Patient Outcome Assessment"[Majr:NoExp] OR "Patient-Centered Care"[Mesh] OR "Patient Satisfaction"[Mesh] OR “patient-cent* outcome*”[tw] OR “patient-cent* measure*”[tw]OR “patient-cent* outcome measure*”[tw] OR “patient cent* outcome*”[tw] OR “patient cent* measure*”[tw] OR “patient cent* outcome measure*”[tw] OR “patient-reported outcome*”[tw] OR “patient-reported measure*”[tw] OR “patient-reported outcome measure*”[tw] OR “patient reported outcome*”[tw] OR “patient reported measure*”[tw] OR “patient reported outcome measure*”[tw] OR “patient-related outcome*”[tw] OR “patient-related measure*”[tw] OR “patient-related outcome measure*”[tw] OR “patient related outcome*”[tw] OR “patient related measure*”[tw] OR “patient related outcome measure*”[tw] OR “patient-cent* care”[tw] OR “patient cent* care”[tw] OR “patient opinion*”[tw] OR PROM*[tw] OR PREM*[tw] OR “patient-reported assessment*”[tw] OR “self-reported measure*”[tw] OR “symptom score*”[tw] OR “symptom scale*”[tw] OR “patient questionnaire*”[tw] OR “patient survey*”[tw] OR “patient satisf*”[tw] OR “patient perspect*”[tw] OR “patient experien*”[tw] OR “patient engag*”[tw] OR “patient-reported experience measure*”[tw] OR “patient reported experience measure*”[tw] OR “patient-reported experience outcome*”[tw] OR “patient reported experience outcome*”[tw] OR “patient-reported experience outcome measure*”[tw] OR “patient reported experience outcome measure*”[tw] OR “patient-cent* experience measure*”[tw] OR “patient cent* experience measure*”[tw] OR “patient-cent* experience outcome*”[tw] OR “patient cent* experience outcome*”[tw] OR “patient-cent* experience outcome measure*”[tw] OR “patient cent* experience outcome measure*”[tw] OR “patient-related experience outcome*”[tw] OR “patient related experience outcome*”[tw] OR “patient-related experience measure*”[tw] OR “patient related experience measure*”[tw] OR “patient-related experience outcome measure*”[tw] OR “patient related experience outcome measure*”[tw]

AND

"Respiratory Insufficiency"[Majr:NoExp] OR "Dyspnea"[Majr:NoExp] OR "Hypoxia"[Majr:NoExp] OR "Tachypnea"[Mesh] OR “short* of breath”[tw] OR breathless*[tw] OR dyspn*[tw] OR dyspnea[tw] OR wheez*[tw] OR hypoxi*[tw] OR “respiratory failure”[tw] OR “respiratory extremis”[tw] OR “acute respiratory distress”[tw] OR “respiratory distress”[tw] OR “respiratory insufficiency”[tw] OR “acute hypoxemic respiratory failure”[tw] OR “respiratory compromise”[tw] OR “acute respiratory illness”[tw] OR hypoxemia[tw] OR hypoxaemia[tw] OR “increased respiratory effort”[tw] OR “accessory musc*”[tw] OR “paradoxical breathing”[tw] OR “thoracoabdominal asynchrony”[tw] OR tachypn*[tw]

AND

"Noninvasive Ventilation"[Mesh] OR "Positive-Pressure Respiration"[Mesh] OR “Oxygen Inhalation Therapy”[Mesh] OR “High flow oxygen”[tw] OR HFO[tw] OR “high flow nasal oxygen”[tw] OR HFNO[tw] OR “nasal high flow oxygen”[tw] OR NHFO[tw] OR “continuous positive airway pressure”[tw] OR “bilevel positive airway pressure”[tw] OR “non-invasive ventilation”[tw] OR NIV[tw] OR “respiratory support”[tw] OR “non-invasive respiratory support”[tw] OR “non-invasive positive pressure ventilation” [tw] OR NPPV[tw] OR “bilevel ventilation”[tw] OR BPAP[tw] OR “high-flow nasal cannula”[tw] OR HFNC[tw]

AND

"Emergency Service, Hospital"[Mesh] OR "Emergency Medical Services"[Mesh] OR "Emergency Medicine"[Mesh] OR "Hospitals"[Mesh] OR "Critical Care"[Mesh] OR "Intensive Care Units"[Mesh] OR "Hospital Medicine"[Mesh] OR "Respiratory Care Units"[Mesh] OR "Clinical Observation Units"[Mesh] OR “acute setting”[tw] OR “acute ward”[tw] OR “acute hospital”[tw] OR “Acute medical unit”[tw] OR AMU[tw] OR “Acute assessment unit”[tw] OR AAU[tw] OR “Emergency department”[tw] OR ED[tw] OR “emergency assessment unit”[tw] OR EAU[tw] OR “emergency cent*”[tw] OR “emergency ward”[tw] OR “emergency room”[tw] OR ER[tw] OR “accident and emergency”[tw] OR A&E[tw] OR “emergency medicine”[tw] OR EM[tw] OR “emergency care”[tw] OR “Same Day Emergency Care”[tw] OR SDEC[tw] OR “Short stay unit”[tw] OR SSU[tw] OR “Clinical decision unit”[tw] OR CDU[tw] OR “High* dependency unit”[tw] OR HDU[tw] OR “Higher monitoring unit”[tw] OR HMU[tw] OR “urgent care”[tw] OR “urgent care cent*”[tw] OR “intensive care unit”[tw] OR ICU[tw] OR “critical care unit”[tw] OR CCU[tw] OR “intensive treatment unit”[tw] OR ITU[tw] OR “critical care”[tw] OR “intensive care medicine”[tw] OR ICM[tw] OR “acute care”[tw] OR “inpatient”[tw] OR “hospital”[tw] OR “Hospitalised”[tw] OR “hospitalized”[tw] OR “ward”[tw] OR “medical assessment unit”[tw] OR MAU[tw] OR “intensive therapy unit”[tw] OR “acute hospital”[tw] OR “emergency hospital”[tw] OR “respiratory ward”[tw] OR “acute respiratory ward”[tw] OR “emergency respiratory ward”[tw] OR “respiratory unit”[tw] OR “acute respiratory unit”[tw] OR “emergency respiratory unit”[tw] OR “respiratory support unit”[tw] OR RSU[tw] OR “hospital emergency service”[tw] OR “emergency unit”[tw]

AND

"Adult"[Mesh] OR adult*[tw] OR elderly[tw] OR “older person”[tw] OR aged[tw] OR “middle age*”[tw] OR “grown up”[tw] OR “grown-up”[tw] OR “fully-grown”[tw]

Search filters: English, Human

**Web of Science (Core Collection):**

“patient-cent* outcome*” OR “patient-cent* measure*”OR “patient-cent* outcome measure*” OR “patient cent* outcome*” OR “patient cent* measure*” OR “patient cent* outcome measure*” OR “patient-reported outcome*” OR “patient-reported measure*” OR “patient-reported outcome measure*” OR “patient reported outcome*” OR “patient reported measure*” OR “patient reported outcome measure*” OR “patient-related outcome*” OR “patient-related measure*” OR “patient-related outcome measure*” OR “patient related outcome*” OR “patient related measure*” OR “patient related outcome measure*” OR “patient-cent* care” OR “patient cent* care” OR “patient opinion*” OR PROM* OR PREM* OR “patient-reported assessment*” OR “self-reported measure*” OR “symptom score*” OR “symptom scale*” OR “patient questionnaire*” OR “patient survey*” OR “patient satisf*” OR “patient perspect*” OR “patient experien*” OR “patient engag*” OR “patient-reported experience measure*” OR “patient reported experience measure*” OR “patient-reported experience outcome*” OR “patient reported experience outcome*” OR “patient-reported experience outcome measure*” OR “patient reported experience outcome measure*” OR “patient-cent* experience measure*” OR “patient cent* experience measure*” OR “patient-cent* experience outcome*” OR “patient cent* experience outcome*” OR “patient-cent* experience outcome measure*” OR “patient cent* experience outcome measure*” OR “patient-related experience outcome*” OR “patient related experience outcome*” OR “patient-related experience measure*” OR “patient related experience measure*” OR “patient-related experience outcome measure*” OR “patient related experience outcome measure*”

AND

“short* of breath” OR breathless* OR dyspn* OR wheez* OR hypoxi* OR “respiratory failure” OR “respiratory extremis” OR “acute respiratory distress” OR “respiratory distress” OR “respiratory insufficiency” OR “acute hypoxemic respiratory failure” OR “respiratory compromise” OR “acute respiratory illness” OR hypoxemia OR hypoxaemia OR “increased respiratory effort” OR “accessory musc*” OR “paradoxical breathing” OR “thoracoabdominal asynchrony” OR tachypn*

AND

“High flow oxygen” OR HFO OR “high flow nasal oxygen” OR HFNO OR “nasal high flow oxygen” OR NHFO OR “continuous positive airway pressure” OR “bilevel positive airway pressure” OR *PAP OR “non-invasive ventilation” OR NIV OR “respiratory support” OR “non-invasive respiratory support” OR “non-invasive positive pressure ventilation” OR NPPV OR “bilevel ventilation” OR BPAP OR “high-flow nasal cannula” OR HFNC

AND

“acute setting” OR “acute ward” OR “acute hospital” OR “Acute medical unit” OR AMU OR “Acute assessment unit” OR AAU OR “Emergency department” OR ED OR “emergency assessment unit” OR EAU OR “emergency cent*” OR “emergency ward” OR “emergency room” OR ER OR “accident and emergency” OR A&E OR “emergency medicine” OR EM OR “emergency care” OR “Same Day Emergency Care” OR SDEC OR “Short stay unit” OR SSU OR “Clinical decision unit” OR CDU OR “High* dependency unit” OR HDU OR “High* monitoring unit” OR HMU OR “Urgent assessment unit” OR UAU OR “urgent care” OR “urgent care cent*” OR “intensive care unit” OR ICU OR “critical care unit” OR CCU OR “intensive treatment unit” OR ITU OR “critical care” OR “intensive care medicine” OR ICM OR “acute care” OR “inpatient” OR “hospital” OR “Hospitalised” OR “hospitalized” OR “ward” OR “medical assessment unit” OR MAU OR “intensive therapy unit” OR “acute hospital” OR “emergency hospital” OR “respiratory ward” OR “acute respiratory ward” OR “emergency respiratory ward” OR “respiratory unit” OR “acute respiratory unit” OR “emergency respiratory unit” OR “respiratory support unit” OR RSU OR “hospital emergency service” OR “emergency unit”

AND

adult* OR elderly OR “older person” OR aged OR “middle age*” OR “grown up” OR “grown-up” OR “fully-grown”

Core collection

Search field: topic

Search filters: English

**Scopus:**

TITLE-ABS-KEY(

( "patient-cent* outcome*" OR "patient-cent* measure*" OR "patient-cent* outcome measure*" OR "patient cent* outcome*" OR "patient cent* measure*" OR "patient cent* outcome measure*" OR "patient reported outcome*" OR "patient reported measure*" OR "patient reported outcome measure*" OR "patient-reported outcome*" OR "patient-reported measure*" OR "patient-reported outcome measure*" OR "patient related outcome*" OR "patient related measure*" OR “patient related outcome measure*” OR "patient-related outcome*" OR "patient-related measure*" OR “patient-related outcome measure*” OR "patient satisf*" OR "patient perspect*" OR "patient experien*" OR "patient engag*" OR "patient-cent* care" OR "patient cent* care" OR PROM* OR PREM* OR "patient-reported assessment*" OR "self-reported measure*" OR "symptom score*" OR "symptom scale*" OR "patient questionnaire*" OR "patient survey*" OR "patient-reported experience measure*" OR "patient reported experience measure*" OR “patient-reported experience outcome*” OR “patient reported experience outcome*” OR “patient-reported experience outcome measure*” OR “patient reported experience outcome measure*” OR “patient-cent* experience measure*” OR “patient cent* experience measure*” OR “patient-cent* experience outcome*” OR “patient cent* experience outcome*” OR “patient-cent* experience outcome measure*” OR “patient cent* experience outcome measure*” OR “patient-related experience outcome*” OR “patient related experience outcome*” OR “patient-related experience measure*” OR “patient related experience measure*” OR “patient-related experience outcome measure*” OR “patient related experience outcome measure*”)

AND

( "respiratory insufficiency" OR "respiratory failure" OR "respiratory distress" OR "acute respiratory distress" OR "acute respiratory illness" OR "respiratory compromise" OR "respiratory extremis" OR "acute hypoxemic respiratory failure" OR breathless* OR dyspnea OR dyspnoea OR wheez* OR hypoxi*

OR "short* of breath" OR hypoxemia OR hypoxaemia OR “increased respiratory effort” OR “accessory musc*” OR “paradoxical breathing” OR “thoracoabdominal asynchrony” OR tachypn*)

AND

( "high flow oxygen" OR "high flow nasal oxygen" OR "nasal high flow oxygen" OR HFO OR HFNO OR NHFO OR "continuous positive airway pressure" OR "bilevel positive airway pressure" OR "non-invasive ventilation" OR NIV OR "respiratory support" OR "non-invasive respiratory support" OR "non-invasive positive pressure ventilation" OR NPPV OR "bilevel ventilation" OR BPAP OR "high-flow nasal cannula" OR HFNC)

AND

( "acute setting" OR "acute ward" OR "acute hospital" OR "acute medical unit" OR AMU OR "acute assessment unit" OR AAU OR "emergency department" OR ED OR "emergency assessment unit" OR EAU

OR "emergency cent*" OR "emergency ward" OR "emergency room" OR ER OR "accident and emergency" OR "A&E" OR "emergency medicine" OR EM OR "emergency care" OR "same day emergency care" OR SDEC OR "short stay unit" OR SSU OR "clinical decision unit" OR CDU OR "high* dependency unit" OR HDU OR "higher monitoring unit" OR HMU OR "urgent care" OR "urgent care cent*" OR "intensive care unit" OR ICU OR "critical care unit" OR CCU OR "intensive treatment unit" OR ITU OR "critical care" OR "intensive care medicine" OR ICM OR "acute care" OR inpatient OR hospital OR hospitalised OR hospitalized OR ward OR “medical assessment unit” OR MAU OR “intensive therapy unit” OR “acute hospital” OR “emergency hospital” OR “respiratory ward” OR “acute respiratory ward” OR “emergency respiratory ward” OR “respiratory unit” OR “acute respiratory unit” OR “emergency respiratory unit” OR “respiratory support unit” OR RSU OR “hospital emergency service” OR “emergency unit”)

AND

( adult* OR elderly OR "older person" OR aged OR "middle age*" OR "grown up" OR "grown-up" OR "fully-grown"))

Search limits: English, human, adult

**Cochrane CENTRAL:**

MeSH descriptor: [Patient Reported Outcome Measures] explode all trees OR MeSH descriptor: [Patient Outcome Assessment] explode all trees OR MeSH descriptor: [Patient-Centered Care] explode all trees OR MeSH descriptor: [Patient Satisfaction] explode all trees OR “patient centred outcome” OR “patient centered outcome” OR “patient centred outcomes” OR “patient centered outcomes” OR “patient centered measure” OR “patient centred measure” OR “patient centered measures” OR “patient centred measures” OR “patient centered outcome measure” OR “patient centered outcome measures” OR “patient centred outcome measure” OR “patient centred outcome measures” OR “patient reported outcome” OR “patient reported outcomes” OR “patient reported measure” OR “patient reported measures” OR “patient reported outcome measure” OR “patient reported outcome measures” OR “patient related outcome” OR “patient related outcomes” OR “patient related measure” OR “patient related measures” OR “patient related outcome measure” OR “patient related outcome measures” OR “patient satisfaction” OR “patient perspective” OR “patient perspectives” OR “patient experience” OR “patient experiences” OR “patient engagement” OR “patient centered care” OR “patient centred care” OR “patient opinion” OR “patient opinions” OR PROM* OR PREM* OR “patient reported assessment” OR “patient reported assessments” OR “symptom score” OR “symptom scale” OR “symptom scores” OR “symptom scales” OR “patient questionnaire” OR “patient survey” OR “patient questionnaires” OR “patient surveys” OR “patient reported experience outcome” OR “patient reported experience outcomes” OR “patient reported experience measure” OR “patient reported experience measures” OR “patient reported experience outcome measure” OR “patient reported experience outcome measures” OR “patient related experience outcome” OR “patient related experience outcomes” OR “patient related experience measure” OR “patient related experience measures” OR “patient related experience outcome measure” OR “patient related experience outcome measures” OR “patient centred experience measure” OR “patient centred experience measures” OR “patient centered experience measure” OR “patient centered experience measures” OR “patient centred experience outcome” OR “patient centred experience outcomes” OR “patient centered experience outcome” OR “patient centered experience outcomes” OR “patient centred experience outcome measure” OR “patient centred experience outcome measures” OR “patient centered experience outcome measure” OR “patient centered experience outcome measures”

AND

MeSH descriptor: [Respiratory Insufficiency] explode all trees OR MeSH descriptor: [Dyspnea] explode all trees OR MeSH descriptor: [Hypoxia] explode all trees OR MeSH descriptor: [Tachypnea] explode all trees OR “short of breath” OR “shortness of breath” OR breathless* OR dyspnoea OR dyspnea OR wheez* OR hypoxi* OR “respiratory failure” OR “respiratory extremis” OR “acute respiratory distress” OR “respiratory distress” OR “respiratory insufficiency” OR “acute hypoxemic respiratory failure” OR “respiratory compromise” OR “acute respiratory illness” OR hypoxemia OR hypoxaemia OR “increased respiratory effort” OR “accessory muscle use” OR “accessory musculature use” OR “paradoxical breathing” OR “thoracoabdominal asynchrony” OR tachypn*

AND

MeSH descriptor: [Noninvasive Ventilation] explode all trees OR MeSH descriptor: [Positive-Pressure Respiration] explode all trees OR MeSH descriptor: [Oxygen Inhalation Therapy] explode all trees OR “High flow oxygen” OR HFO OR “high flow nasal oxygen” OR HFNO OR “nasal high flow oxygen” OR NHFO OR “continuous positive airway pressure” OR “bilevel positive airway pressure” OR *PAP OR “non invasive ventilation” OR NIV OR “respiratory support” OR “non invasive respiratory support” OR “non invasive positive pressure ventilation” OR NPPV OR “bilevel ventilation” OR BPAP OR “high flow nasal cannula” OR HFNC

AND

MeSH descriptor: [Emergency Service, Hospital] explode all trees OR MeSH descriptor: [Emergency Medical Services] explode all trees OR MeSH descriptor: [Emergency Medicine] explode all trees OR MeSH descriptor: [Hospitals] explode all trees OR MeSH descriptor: [Critical Care] explode all trees OR MeSH descriptor: [Intensive Care Units] explode all trees OR MeSH descriptor: [Hospital Medicine] explode all trees OR MeSH descriptor: [Respiratory Care Units] explode all trees OR MeSH descriptor: [Clinical Observation Units] explode all trees OR “acute setting” OR “acute ward” OR “acute hospital” OR “Acute medical unit” OR AMU OR “Acute assessment unit” OR AAU OR “Emergency department” OR ED OR “emergency assessment unit” OR EAU OR “emergency centre” OR “emergency center” OR “emergency ward” OR “emergency room” OR ER OR “accident and emergency” OR A&E OR “emergency medicine” OR EM OR “emergency care” OR “Same Day Emergency Care” OR SDEC OR “Short stay unit” OR SSU OR “Clinical decision unit” OR CDU OR “High dependency unit” OR “Higher dependency unit” OR HDU OR “High monitoring unit” OR “Higher monitoring unit” OR HMU OR “Urgent assessment unit” OR UAU OR “urgent care” OR “urgent care center” OR “urgent care centre” OR “intensive care unit” OR ICU OR “critical care unit” OR CCU OR “intensive treatment unit” OR ITU OR “critical care” OR “intensive care medicine” OR ICM OR “acute care” OR “inpatient” OR “hospital” OR “Hospitalised” OR “hospitalized” OR “ward” OR “medical assessment unit” OR MAU OR “intensive therapy unit” OR “acute hospital” OR “emergency hospital” OR “respiratory ward” OR “acute respiratory ward” OR “emergency respiratory ward” OR “respiratory unit” OR “acute respiratory unit” OR “emergency respiratory unit” OR “respiratory support unit” OR RSU OR “hospital emergency service” OR “emergency unit”

AND

MeSH descriptor: [Adult] this term only OR adult* OR elderly OR “older person” OR aged OR “middle age” OR “middle aged” OR “grown up” OR “fully grown”

**CINAHL Ultimate (EBSCO):**

“patient-cent* outcome*” OR “patient-cent* measure*”OR “patient-cent* outcome measure*” OR “patient cent* outcome*” OR “patient cent* measure*” OR “patient cent* outcome measure*” OR “patient-reported outcome*” OR “patient-reported measure*” OR “patient-reported outcome measure*” OR “patient reported outcome*” OR “patient reported measure*” OR “patient reported outcome measure*” OR “patient-related outcome*” OR “patient-related measure*” OR “patient-related outcome measure*” OR “patient related outcome*” OR “patient related measure*” OR “patient related outcome measure*” OR “patient-cent* care” OR “patient cent* care” OR “patient opinion*” OR PROM* OR PREM* OR “patient-reported assessment*” OR “self-reported measure*” OR “symptom score*” OR “symptom scale*” OR “patient questionnaire*” OR “patient survey*” OR “patient satisf*” OR “patient perspect*” OR “patient experien*” OR “patient engag*” OR “patient-reported experience measure*” OR “patient reported experience measure*” OR “patient-reported experience outcome*” OR “patient reported experience outcome*” OR “patient-reported experience outcome measure*” OR “patient reported experience outcome measure*” OR “patient-cent* experience measure*” OR “patient cent* experience measure*” OR “patient-cent* experience outcome*” OR “patient cent* experience outcome*” OR “patient-cent* experience outcome measure*” OR “patient cent* experience outcome measure*” OR “patient-related experience outcome*” OR “patient related experience outcome*” OR “patient-related experience measure*” OR “patient related experience measure*” OR “patient-related experience outcome measure*” OR “patient related experience outcome measure*”

AND

“short* of breath” OR breathless* OR dyspnoea OR dyspnea OR wheez* OR hypoxi* OR “respiratory failure” OR “respiratory extremis” OR “acute respiratory distress” OR “respiratory distress” OR “respiratory insufficiency” OR “acute hypoxemic respiratory failure” OR “respiratory compromise” OR “acute respiratory illness” OR hypoxemia OR hypoxaemia OR “increased respiratory effort” OR “accessory musc*” OR “paradoxical breathing” OR “thoracoabdominal asynchrony” OR tachypn*

AND

“High flow oxygen” OR HFO OR “high flow nasal oxygen” OR HFNO OR “nasal high flow oxygen” OR NHFO OR “continuous positive airway pressure” OR “bilevel positive airway pressure” OR *PAP OR “non-invasive ventilation” OR NIV OR “respiratory support” OR “non-invasive respiratory support” OR “non invasive positive pressure ventilation” OR NPPV OR “bilevel ventilation” OR BPAP OR “high flow nasal cannula” OR HFNC

AND

“acute setting” OR “acute ward” OR “acute hospital” OR “Acute medical unit” OR AMU OR “Acute assessment unit” OR AAU OR “Emergency department” OR ED OR “emergency assessment unit” OR EAU OR “emergency cent*” OR “emergency ward” OR “emergency room” OR ER OR “accident and emergency” OR A&E OR “emergency medicine” OR EM OR “emergency care” OR “Same Day Emergency Care” OR SDEC OR “Short stay unit” OR SSU OR “Clinical decision unit” OR CDU OR “High* dependency unit” OR HDU OR “High* monitoring unit” OR HMU OR “Urgent assessment unit” OR UAU OR “urgent care” OR “urgent care cent*” OR “intensive care unit” OR ICU OR “critical care unit” OR CCU OR “intensive treatment unit” OR ITU OR “critical care” OR “intensive care medicine” OR ICM OR “acute care” OR “inpatient” OR “hospital” OR “Hospitalised” OR “hospitalized” OR “ward” OR “medical assessment unit” OR MAU OR “intensive therapy unit” OR “acute hospital” OR “emergency hospital” OR “respiratory ward” OR “acute respiratory ward” OR “emergency respiratory ward” OR “respiratory unit” OR “acute respiratory unit” OR “emergency respiratory unit” OR “respiratory support unit” OR RSU OR “hospital emergency service” OR “emergency unit”

AND

adult* OR elderly OR “older person” OR aged OR “middle age*” OR “grown up” OR “grown-up” OR “fully-grown”

Search Limiters: English Language, Human

Search Expanders: Apply related words, Apply equivalent subjects

Search modes: Find any of my search terms

**Embase (Ovid):**

"patient-cent* outcome*" OR "patient-cent* measure*" OR "patient-cent* outcome measure*" OR "patient cent* outcome*" OR "patient cent* measure*" OR "patient cent* outcome measure*" OR "patient-reported outcome*" OR "patient-reported measure*" OR "patient-reported outcome measure*" OR "patient reported outcome*" OR "patient reported measure*" OR "patient reported outcome measure*" OR "patient-related outcome*" OR "patient-related measure*" OR "patient-related outcome measure*" OR "patient related outcome*" OR "patient related measure*" OR "patient related outcome measure*" OR "patient satisf*" OR "patient perspect*" OR "patient experien*" OR "patient engag*" OR "patient-cent* care" OR "patient cent* care" OR "patient opinion*" OR PROM* OR PREM* OR "patient-reported assessment*" OR "self-reported measure*" OR "symptom score*" OR "symptom scale*" OR "patient questionnaire*" OR "patient survey*" OR "patient-reported experience outcome*" OR "patient reported experience outcome*" OR "patient-reported experience measure*" OR "patient reported experience measure*" OR "patient-reported experience outcome measure*" OR "patient reported experience outcome measure*" OR "patient-related experience outcome*" OR "patient related experience outcome*" OR "patient-related experience measure*" OR "patient related experience measure*" OR "patient-related experience outcome measure*" OR "patient related experience outcome measure*" OR "patient-cent* experience measure*" OR "patient cent* experience outcome measure*" OR "patient-cent* experience outcome*" OR "patient cent* experience outcome*" OR "patient-cent* experience outcome measure*" OR "patient cent* experience outcome measure*"

AND

"short* of breath" OR breathless* OR dyspnoea OR dyspnea OR wheez* OR hypoxi* OR "respiratory failure" OR "respiratory extremis" OR "acute respiratory distress" OR "respiratory distress" OR "respiratory insufficiency" OR "acute hypoxemic respiratory failure" OR "respiratory compromise" OR "acute respiratory illness" OR hypoxemia OR hypoxaemia OR "increased respiratory effort" OR "accessory musc*" OR "paradoxical breathing" OR "thoracoabdominal asynchrony" OR tachypn*

AND

"High flow oxygen" OR HFO OR "high flow nasal oxygen" OR HFNO OR "nasal high flow oxygen" OR NHFO OR "continuous positive airway pressure" OR "bilevel positive airway pressure" OR "non-invasive ventilation" OR NIV OR "respiratory support" OR "non-invasive respiratory support" OR "non-invasive positive pressure ventilation" OR NPPV OR "bilevel ventilation" OR BPAP OR "high-flow nasal cannula" OR HFNC

AND

"acute setting" OR "acute ward" OR "acute hospital" OR "Acute medical unit" OR AMU OR "Acute assessment unit" OR AAU OR "Emergency department" OR ED OR "emergency assessment unit" OR EAU OR "emergency cent*" OR "emergency ward" OR "emergency room" OR ER OR "accident and emergency" OR A&E OR "emergency medicine" OR EM OR "emergency care" OR "Same Day Emergency Care" OR SDEC OR "Short stay unit" OR SSU OR "Clinical decision unit" OR CDU OR "High* dependency unit" OR HDU OR "High* monitoring unit" OR HMU OR "Urgent assessment unit" OR UAU OR "urgent care" OR "urgent care cent*" OR "intensive care unit" OR ICU OR "critical care unit" OR CCU OR "intensive treatment unit" OR ITU OR "critical care" OR "intensive care medicine" OR ICM OR "acute care" OR inpatient OR hospital OR hospitalised OR hospitalized OR ward OR "medical assessment unit" OR MAU OR "intensive therapy unit" OR "acute hospital" OR "emergency hospital" OR "respiratory ward" OR "acute respiratory ward" OR "emergency respiratory ward" OR "respiratory unit" OR "acute respiratory unit" OR "emergency respiratory unit" OR "respiratory support unit" OR RSU OR "hospital emergency service" OR "emergency unit"

AND

adult* OR elderly OR "older person" OR aged OR "middle age*" OR "grown up" OR "grown-up" OR "fully-grown"

Search limits: human, english

**PsycINFO (Ovid):**

((patient reported outcome* or patient reported measure* or patient reported outcome measure* or patient-reported outcome* or patient-reported measure* or patient-reported outcome measure* or patient related outcome* or patient related measure* or patient related outcome measure* or patient-related outcome* or patient-related measure* or patient-related outcome measure* or patient-cent* outcome* or patient-cent* measure* or patient-cent* outcome measure* or patient cent* outcome* or patient cent* measure* or patient cent* outcome measure* or patient satisf* or patient perspect* or patient experien* or patient engag* or patient-cent* care or patient cent* care or PROM* or PREM* or patient-reported assessment* or self-reported measure* or symptom score* or symptom scale* or patient questionnaire* or patient survey* or patient-reported experience measure* or patient reported experience measure* or patient-reported experience outcome* or patient reported experience outcome* or patient-reported experience outcome measure* or patient reported experience outcome measure* or patient-related experience measure* or patient related experience measure* or patient-related experience outcome* or patient related experience outcome* or patient-related experience outcome measure* or patient related experience outcome measure* or patient-cent* experience measure* or patient cent* experience measure* or patient- cent* experience outcome* or patient cent* experience outcome* or patient- cent* experience outcome measure* or patient cent* experience outcome measure*)

AND

(respiratory insufficiency or respiratory failure or respiratory distress or acute respiratory distress or acute respiratory illness or respiratory compromise or respiratory extremis or acute hypoxemic respiratory failure or breathless* or dyspnea or dyspnoea or wheez* or hypoxi* or short* of breath or hypoxemia or hypoxaemia or increased respiratory effort or accessory musc* or paradoxical breathing or thoracoabdominal asynchrony or tachypn*)

AND
(high flow oxygen or high flow nasal oxygen or nasal high flow oxygen or HFO or HFNO or NHFO or continuous positive airway pressure or bilevel positive airway pressure CPAP or BiPAP or non-invasive ventilation or NIV or respiratory support or non-invasive respiratory support or non-invasive positive pressure ventilation or NPPV or bilevel ventilation or BPAP or high-flow nasal cannula or HFNC)

AND

(acute setting or acute ward or acute hospital or acute medical unit or AMU or acute assessment unit or AAU or emergency department or ED or emergency assessment unit or EAU or emergency cent* or emergency ward or emergency room or ER or accident) and emergency) or A&E or emergency medicine or EM or emergency care or same day emergency care or SDEC or short stay unit or SSU or clinical decision unit or CDU or high* dependency unit or HDU or higher monitoring unit or HMU or urgent care or urgent care cent* or intensive care unit or ICU or critical care unit or CCU or intensive treatment unit or ITU or critical care or intensive care medicine or ICM or acute care or inpatient or hospital or hospitalised or hospitalized or ward or medical assessment unit or MAU or intensive therapy unit or acute hospital or emergency hospital or respiratory ward or acute respiratory ward or emergency respiratory ward or respiratory unit or acute respiratory unit or emergency respiratory unit or respiratory support unit or RSU or hospital emergency service or emergency unit)

AND

(adult* or elderly or older person or aged or middle age* or grown up or grown-up or fully-grown)).af.

APA PsycInfo 1806 - present

Search limits: English, human

**Google Scholar:**

("patient cent* outcome measure*" OR “patient reported measure” OR “patient reported outcome measure*” OR "patient satisf*" OR "patient perspective*" OR "patient experience*" OR "patient engagement*" OR PROM* OR PREM* OR "self reported measure*" OR "patient reported experience measure*")

AND

("respiratory failure" OR "acute respiratory distress" OR "respiratory extremis" OR breathless* OR dyspn* OR wheez* OR hypox* OR "short* of breath" OR tachypn*) AND ( "high flow oxygen" OR "nasal high flow oxygen" OR HFO OR HFNO OR NHFO OR "positive airway pressure" OR "non-invasive ventilation" OR NIV OR "non-invasive respiratory support" OR NPPV OR "bilevel ventilation" OR *PAP OR "high flow nasal cannula" OR HFNC)

AND

("acute setting" OR "acute ward" OR "acute hospital" OR "acute medical unit" OR "acute assessment unit" OR "emergency department" OR ED OR "emergency assessment unit" OR "emergency cent*" OR "emergency ward" OR "emergency room" OR "accident and emergency" OR "emergency care" OR "same day emergency care" OR "short stay unit" OR "clinical decision unit" OR "high* dependency unit" OR "high* monitoring unit" OR "urgent care" OR "intensive care unit" OR "critical care unit" OR "intensive treatment unit" OR "critical care" OR "acute care" OR inpatient OR “medical assessment unit” OR “intensive therapy unit” OR “acute hospital” OR “emergency hospital” OR “respiratory ward” OR “acute respiratory ward” OR “respiratory unit” OR “acute respiratory unit” OR “respiratory support unit” OR “emergency unit”)

AND

(adult* OR elderly OR "old* person" OR "middle age*")

AND

(thesis OR dissertation OR conference OR report)

Search filters: English language

Format: Find articles

-	with all of the words

-	where my words occur: anywhere in the article

*NB: a 256 character limit exists on all Google Scholar searches*

### **Appendix II: Data extraction table**

|  | Title | Author(s) | Year of publication | Geogr. location | Journal | Study funding source | Reference citation | Study design | Clinical setting | Study aims | Study duration | | Study definition of acute respiratory failure | Inclusion criteria | Exclusion criteria | Control | Intervention | Outcome measures | Length of follow up of study | … |
| --- | --- | --- | --- | --- | --- | --- | --- | --- | --- | --- | --- | --- | --- | --- | --- | --- | --- | --- | --- | --- |
|  |  |  |  |  |  |  |  |  |  |  | Start date | End date |  |  |  | Control group | Intervention group |  |  |  |
| 1 |  |  |  |  |  |  |  |  |  |  |  |  |  |  |  |  |  |  |  | … |
| 2 |  |  |  |  |  |  |  |  |  |  |  |  |  |  |  |  |  |  |  | … |
| 3 |  |  |  |  |  |  |  |  |  |  |  |  |  |  |  |  |  |  |  | … |

| … | Withdrawals &  Exclusions | Participants & sample size | | Key findings of study | PRM(s) Importance (1*/2*/3* outcome) | PRM(s) studied & method of collection (paper, verbal, electronic etc.) | Study description of PRM | Role of individual who used/ measured PRM | Point(s) at which PRM is used in patient journey | Length of follow up of PRM | Does the study report the PRM is validated | Study detail on the PRM development process - were patients involved? | Key findings relating to PRM(s) | Notes |
| --- | --- | --- | --- | --- | --- | --- | --- | --- | --- | --- | --- | --- | --- | --- |
|  |  | Control | Intervention |  |  |  |  |  |  |  |  |  |  |  |
| … |  |  |  |  |  |  |  |  |  |  |  |  |  |  |
| … |  |  |  |  |  |  |  |  |  |  |  |  |  |  |
| … |  |  |  |  |  |  |  |  |  |  |  |  |  |  |
